# Combined Effects of Different Endocrine-Disrupting Chemicals (EDCs) on Prostate Gland

**DOI:** 10.3390/ijerph18189772

**Published:** 2021-09-16

**Authors:** Maria De Falco, Vincenza Laforgia

**Affiliations:** 1 Department of Biology, University of Naples ‘‘Federico II’’, 80126 Naples, Italy; vincenza.laforgia@unina.it; 2 National Institute of Biostructures and Biosystems (INBB), 00136 Rome, Italy; 3 Center for Studies on Bioinspired Agro-Environmental Technology (BAT Center), 80055 Portici, Italy

**Keywords:** endocrine-disrupting chemicals (EDCs), androgens, estrogens, endocrine system, mixture, prostate gland, prostate cancer (PC), benign prostatic hyperplasia (BPH)

## Abstract

Endocrine-disrupting chemicals (EDCs) belong to a heterogeneous class of environmental pollutants widely diffused in different aquatic and terrestrial habitats. This implies that humans and animals are continuously exposed to EDCs from different matrices and sources. Moreover, pollution derived from anthropic and industrial activities leads to combined exposure to substances with multiple mechanisms of action on the endocrine system and correlated cell and tissue targets. For this reason, specific organs, such as the prostate gland, which physiologically are under the control of hormones like androgens and estrogens, are particularly sensitive to EDC stimulation. It is now well known that an imbalance in hormonal regulation can cause the onset of various prostate diseases, from benign prostate hyperplasia to prostate cancer. In this review, starting with the description of normal prostate gland anatomy and embryology, we summarize recent studies reporting on how the multiple and simultaneous exposure to estrogenic and anti-androgenic compounds belonging to EDCs are responsible for an increase in prostate disease incidence in the human population.

## 1. Introduction

Endocrine-disrupting chemicals (EDCs) are exogenous substances (drugs, pesticides, plastic additives, organic pollutants, and natural compounds) able to alter the physiology of the endocrine system by interfering with the effect, biosynthesis, transport, and metabolism of hormones [1,2,3]. It is now well known that, like hormones, EDCs can act at a low dose, may have non-monotonic dose responses, can target specific tissues, can show different effects and dose responses during development in adults, and are likely not to have a threshold [4,5]. They are present in daily products, such as food cans, metals [2,6], industrial chemicals and other chemicals [1,2], perfumes (which may contain phthalates), sunscreen containing parabens and alkylphenols, drugs (containing nonylphenol), flame retardants (which may contain PCBs), recycled paper, plastic bottles (which may contain polycarbonate and bisphenol A (BPA)), lubricants (where nonylphenol may be present), plastics and food packaging, and hospital instruments (which may contain phthalates) [3,7]. Due to the widespread use of these chemicals, they are released in the environment [2,8] and they are then bio-accumulated in living organisms [2,9]. The main route of exposure is oral intake of contaminated water and food [1,2], but there are other possible sources, such as direct contact with or inhalation of polluted air [2,10]. Moreover, it has been demonstrated that EDCs can cross the placenta, causing a vertical transfer from mother to fetus; breast milk can also contain several EDCs that can thus reach newborns [2,6]. Many EDCs are persistent, and regardless of their prohibition in some countries, they are still present in the environment, affecting the entire population [2]. EDC bioaccumulation and persistence allow them to interfere temporarily or permanently with the hormonal signaling pathways in the endocrine system [11]. Most EDCs act like xenoestrogens or antiandrogens [3]. For these reasons, these substances have become a priority for international policymakers and the scientific community [2,12]. Many EDCs have a chemical structure similar to natural endogenous hormones; thus, EDCs can mimic or block different hormonal pathways, therefore affecting development and inducing pathologies later in adult life [2,8,12]. These substances can interfere with the endocrine system at multiple levels, by agonizing or antagonizing the target receptors or by disrupting the synthesis of the hormones or hormonal release, transport, metabolism, and excretion [11,13,14,15,16,17]. Moreover, several studies have demonstrated the ability of these substances to act on hormone metabolizing enzymes [18,19,20] and also act through epigenetic mechanisms, which are particularly useful in understanding how EDC exposure during development can cause adverse effects in adulthood [11,21]. It has been demonstrated that EDCs can interact with sexual receptors, both estrogen (ER) and androgen (AR), and also with other non-nuclear receptors, such as membrane ERs, thyroid and retinoid receptors, non-steroid receptors, and orphan receptors [1,2,8,11]. The US Food and Drug Administration (FDA) identified more than 1800 chemicals that disrupt at least one of three endocrine pathways (estrogen, androgen, and thyroid) [22,23]. In Europe, the European Commission found 320 of 575 chemicals showed evidence or potential evidence for endocrine disruption [22,24]. Several governmental and non-governmental agencies, such as the Endocrine Society, World Health Organization (WHO), UN Environment Programme (UNEP), and American Academy of Pediatrics, documented the serious adverse effects of EDCs on endocrine processes during susceptible periods of human development as well as a long latency period between exposure and disease due to early-life exposure to chemicals [14,22,25,26]. To date, the harmful impact of these endocrine disruptors has been extensively studied, especially regarding reproductive function and disorders [11,27,28,29,30,31,32,33,34,35,36,37]. Moreover, biological and epidemiological studies correlate EDC exposures with several pathologies, such as obesity, metabolic syndrome, and cancer [3,38]. Their carcinogenicity is given to genotoxicity, epigenetic modifications, or immune system alterations. Obviously, hormone-associated cancers such as breast and prostate cancer (PC) are particularly related to EDC exposure [2].

### 1.1. Prostate Gland: Anatomy and Embryology

The prostate is an important accessory gland of the male reproductive system; it secretes a slightly alkaline fluid that in humans usually constitutes roughly 30% of the volume of the semen [39,40]. This gland develops from the pelvic part of the urogenital sinus (UGS), located at the base of the developing urinary bladder [41]. The UGS, responding to androgens, particularly testosterone (T) secreted by fetal testes and 5α-dihydrotestosterone (DHT), branches to form the prostate, due to a coordinated balance between different mechanisms as well as proliferation, adhesion, and migration [41,42]. All these phenomena are mediated by AR [43]. In rats, the prostatic buds appear at embryonic day E18–E19, but most of the prostate branching occurs after the birth [42]. The rodent prostate has three lobes: anterior, dorsolateral, and ventral; the last one has the most extensive branching [42]. Different from rodents, in humans, the prostate morphogenesis develops into a single organ formed by three different parts: the central, peripheral, and transitional zones [41]. Although prostate formation is completed at birth, its functional activity starts at puberty, when the prostate acquires its secretory ability [41,44]. It has been postulated that disease propensity of the prostate with respect to other accessory male organs can derive from its unique embryologic origin [45,46], different from what happens with the seminal vesicle and vas deferens, which arise from the mesodermal Wolffian ducts [45,47,48].

In adults, the prostate is formed by an epithelium that has low proliferation rates, which, in balance with the control of cell death, allow for the maintenance of a constant size of the prostate, although there is a physiological and continuous stimulation by androgens [42]. Indeed, it is well known that a healthy prostate needs a constant amount of androgens, which are essential throughout development [42]. The prostate epithelium is formed by epithelial cells that present all the features of secretory cells: a large endoplasmic reticulum, a well-developed Golgi apparatus, and many secretory granules widely distributed in the cytoplasm. Epithelial cells, in fact, contribute secretions to semen [42]. The prostate gland also contains composite tubule-alveolar glands that are separated from each other through a stromal tissue [42]. This, specifically, is an interstitial tissue formed by different cell types as well as smooth muscle cells, fibroblasts, blood vessels, and nerves [42]. The stromal component, derived from mesenchyma, is equally important, since, working together with prostate epithelium, it helps maintain prostate physiology and contributes to expel secretions to the semen [42,49]. In addition, the proliferation of stromal cells is under the control of high levels of T [42,50]. It has been demonstrated that another important factor in the maintenance and control of prostate size is the epithelial-stromal ratio [42]. Although androgens are essential for prostate growth and function [51], estrogens also play key roles in prostate development, homeostasis, and disease [51,52].

### 1.2. Localization and Expression of Estrogen and Androgen Receptors inside the Prostate Gland

**Figure 1:**
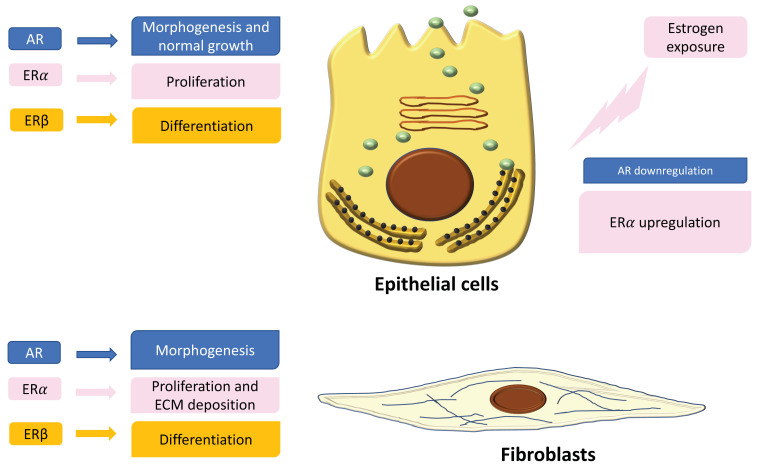
Localization and expression of steroid receptors in epithelial cells and fibroblasts. On the left is shown the role of the receptors in the regulation of cell functions. On the right is shown the different expression of androgen receptors (ARs) and estrogen receptors (ERs) after estrogen exposure during critical windows of exposure.

In the normal prostate, AR is the dominant steroid receptor. All the components of the prostate gland—stromal cells, epithelium, and smooth muscle cells—express androgen receptors [42] (Figure 1). Interaction between AR and its ligand can strongly guide morphogenesis. On the contrary, it has been shown that estrogens act through multiple ERs, including ERα, ERβ, and GPER, which are expressed in different cell types inside the prostate [43]. During development, 17β-estradiol (E2) plays a physiologic role in the modulation of branching morphogenesis through the activation of ERα and in the differentiation of prostate epithelium through ERβ [43,53,54,55,56]. In particular, lower levels of ERα than AR are localized in stromal cells that surround the proximal ducts during early-life prostate morphogenesis [43]. ERα significantly declines with puberty as androgen levels rise, suggesting a specific role during development [43]. Specifically, it has been demonstrated that mouse ERα expressed by different cell types has different actions: fibroblast ERα modulates branching morphogenesis; smooth muscle ERα regulates stromal cell proliferation and deposition of extracellular matrix [43,55,56]. In humans, ERα is expressed by stromal cells during fetal development [43,57,58], and it has been shown that when it is expressed in the periurethral prostatic epithelium during the last gestational period, it is associated with squamous metaplasia [43,58] (Figure 1). Moreover, recently it has been demonstrated that ERα also plays a role in prostatic epithelial stem cells and has involvement in self-renewal and progenitor cell proliferation after estrogen induction [43,59,60,61]. Different from ERα, rodent ERβ is almost exclusively localized in prostate epithelial cells, and it is involved in differentiation processes of the luminal epithelium [43,62]. On the contrary, in humans, ERβ is widely expressed in epithelial and stromal cells by gestational week seven, and it is activated during gestation and for several months after birth, suggesting that ERβ plays a role in development regulation [43,57,58]. Furthermore, ERβ is also localized in stem cells and seems to be involved in progenitor cell differentiation [43,61,63]. Many studies have focused on the central role of steroid receptors in the onset of different prostate pathologies, since it has been shown that they lead to expression and localization changes, initiate growth and differentiation defects during early development, and maintain these phenotypes throughout life [43]. Indeed, in rodents it has been demonstrated that after exposure to high levels of estrogens during the neonatal critical window (post-natal day PND1-5), ERα and AR immediately change, directly driving the early estrogenized phenotype. Specifically, AR protein is sharply downregulated in both stromal and epithelial cells and remains low throughout life, leading to a reduced response to androgens [43] (Figure 1). On the contrary, ERα is upregulated in periductal stromal cells, which in turn permits a transient induction of the prolactin receptor (PRLR) [43]. Different from ERα and AR, ERβ changes later in development or adulthood [43]. Thus, the developing prostate is no longer under AR regulation but is rather driven by several estrogens, through different receptors such as ERα and PRLR. The resulting effect is that programming signals that normally guide development of the prostate are altered, leading to permanent alterations in prostate structure and activity throughout life [43].

### 1.3. The Role of Estrogens in the Prostate Gland

The wide localization and expression of the main steroid receptors (AR, ERα, ERβ) highlight the relevant role of both androgens and estrogens in the control of prostate function and physiology. Indeed, an imbalance in estrogen levels and actions may contribute to aging-associated prostatic disease [45,47]. Several studies have demonstrated that inappropriate estrogen exposure, mainly E2 but also pharmaceutical estrogens and estrogenic EDCs, in terms of dose, type, and timing during prostate development, result in predisposition to an increased disease susceptibility, a phenotype referred to as estrogenic imprinting or developmental estrogenization [43]. Specifically, an altered estrogenic exposure can lead to abnormal growth of the human prostate, with predisposition to diseases such as benign prostatic hyperplasia (BPH) and adenocarcinoma [64]. A Swedish cohort study showed strong correlations between indicators of high levels of pregnancy estradiol (E) and increased risk of prostate cancer [65]. Moreover, African-American men have a twofold higher risk of developing prostate cancer with aging than Caucasian men, and it has been shown that there is a link with elevated maternal estrogens during the first trimester of gestation [66]. Estrogens can increase risk of prostate cancer later in life, since estrogenic compounds are able to reprogram the gland, both structurally and epigenetically, driving differentiation defects [51,66,67,68]. Moreover, estrogen can render the prostate more susceptible to prostate cancer with aging [45,69,70], a concept that reinforces the developmental basis of adult disease paradigm [43]. For this reason, increased concern regarding inappropriate estrogenic exposure has led to attention on EDCs due to their ability to mimic estrogens activating different pathways in the prostate gland.

### 1.4. Prostate Diseases

Prostate diseases, such as prostatitis, enlarged prostate, BPH, and prostate cancer, become very common with age [39]. BPH is prevalent among older men and increases with age; it is found in approximately 70% of men over 60 and up to 90% of men over 80 [71]. BPH develops in the transition zone of the prostate surrounding the proximal urethra, and with the enlargement of the prostate, it may impede urine flow causing a bladder outlet obstruction (BOO), which can be responsible for bothersome lower urinary tract symptoms (LUTS) [71]. LUTS encompass a range of clinical complaints, including weak stream, straining to urinate, incomplete bladder emptying, frequency and urgency of urination, nocturia, and small voided volumes [71,72]. In addition to LUTS, BPH can also lead to other urinary tract complications, such as elevated postvoid residual, urinary retention, bladder diverticula, hydronephrosis, bladder calculi, and renal insufficiency [71,73]. These conditions significantly affect the quality of life of a substantial proportion of men, and the associated healthcare costs are in the billions annually [71,74,75,76]. Prostate cancer (PC) is the most common cancer and is the second leading cause of death for Caucasian men [77,78]. In Europe, about 2.6 million new cases per year are diagnosed; in Italy, 35,000 new cases were estimated through an epidemiological study in 2015 [3,79]. The Western lifestyle seems to play a central role in the etiology of prostate cancer; in fact, western men have an incidence rate up to 15 times greater than Asian men [3]. Moreover, during the last 15 years, the annual incidence rate increased in Korea as well [78,80]. The most well-known risk factors are age, race, family history of prostate cancer, inflammation, and diet, but biologic and experimental evidence support the hypothesis that environmental pollution, particularly the presence of EDCs, can strongly contribute to this increase [78].

Androgens physiologically control growth and functions of the prostate during, but it has been shown that they can also be involved in carcinogenesis [81]. The interaction among androgens and AR promotes prostate cell proliferation by activating AR-responsive genes and pathways in androgen-dependent adult prostate growth [82,83]. For this reason, AR has a major drug target in BPH [83,84] and PC [81,85]. Moreover, it has been demonstrated that estrogens play an important role in male sex hormone secretion as well as in the growth, differentiation, and homeostasis of both normal and cancer prostate cells [81,86,87]. Estrogens have a crucial role in prostate hyperplasia in aging [83,88]. In vivo studies have suggested that the combined administration of estrogen and androgen synergistically induce BPH [71,89]. Specifically, it has been shown that when Wistar rats were treated with T and E2, prostate weight increased at a higher rate than with T treatment alone, together with a higher DNA synthesis index [89,90]. Moreover, it has been demonstrated that in Noble rats, the long-term administration of combined T and E2 induces prostatic carcinoma [89,91,92]. Furthermore, even if mouse prostate is less sensitive to T and E2, it has been observed that combined administration of both T and E2 causes significant glandular prostatic growth accompanied by bladder outlet obstruction in C57BL mice [71,89]. The administration of T + E2 synergistically promoted prostatic growth, and interestingly, this was accompanied by an extremely enlarged bladder, probably due to bladder outlet obstruction [89]. Indeed, it is not important to consider the single amount of androgens or estrogens, but it is necessary to evaluate the ratio of the circulating and intra-prostatic E/T ratio. In elderly men, the E/T ratio is higher than younger men, and it is accompanied by an increase of ER expression, particularly in the stromal compartment [87,93]. The decrease of T is due to a lower production by the testes together with an increase of sex hormone binding globulin levels [81,94]. Moreover, in elderly men there is an increase of free circulating estrogens in the blood. The change of E/T ratio in favor of E may be responsible for the reactivation of cell growth and can induce a subsequent neoplastic transformation [69,81,95]. It has been proposed that estrogen could promote prostate epithelial proliferation through the activation of ERα, a key mediator of cell proliferation [83,96]. An autopsy study revealed that the prevalence of pathological benign lesions, such as hyperplasia, increased markedly in 90% of men older than 80, probably due to ER overexpression [2,81].

### 1.5. EDCs and Prostate Disease

Although risk assessments have been historically conducted on a chemical-by-chemical basis, regulatory agencies are beginning to consider the cumulative risk of chemicals. Moreover, it is now well known that humans [97,98,99,100,101,102,103], fish [11,104,105,106,107,108], and wildlife [109,110,111,112] are continuously exposed to multiple contaminants [103,113]. In this view, it is essential to study the effect of combined multicomponent mixtures on prostate diseases rather than individual substances in order to highlight the involvement of multiple compounds acting simultaneously in prostate pathologies [78,114,115].

**Figure 2:**
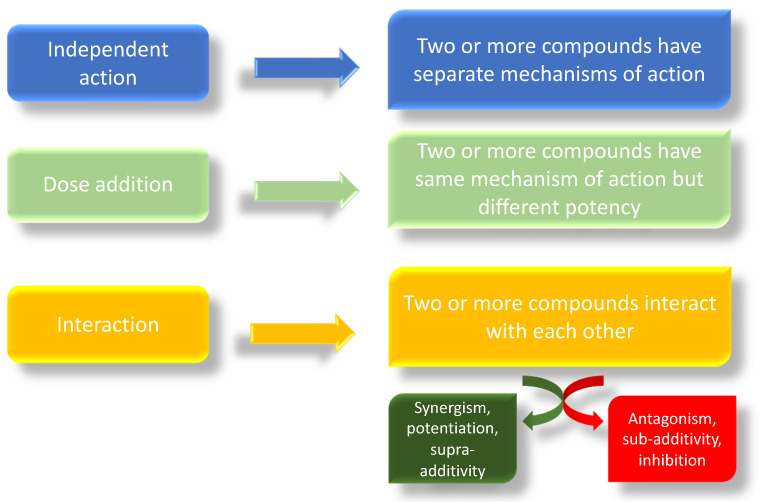
Possible mechanisms of action of combined exposure of different compounds.

Exposure to various EDCs may disrupt the normal androgen and estrogen balance in animals and humans, potentially leading to sex-hormone-sensitive diseases/disorders [1,116,117,118,119,120]. The “something from nothing” principle proposes that exposure to a single chemical may have no observed effects, but exposure to several of these chemicals in a mixture, due to synergistic or additive effects, may be significant [8,99]. These mixtures may even have significant effects at lower concentrations than the “no observed adverse effect levels” (NOAELS) reported for individual chemicals [8,121]. The combined toxicological effects of two or more compounds can take one of three forms: independent action, dose addition, or interaction [113,122,123] (Figure 2). In a mixture, individual compounds may have a single/specific effect due to a separate mechanism of action; in this case we speak of independent action, also known as response addition [113]. In this case, compounds that exhibit dissimilar modes of action can produce different, non-overlapping toxic effects in different organs and systems; thus, it is difficult to identify a combination effect [123]. In the case of simultaneous exposure to several chemicals with different modes of action, the principle of independence of effects is only applicable when all the chemicals in the mixture act through strictly dissimilar modes by affecting strictly different targets (simple dissimilar action). The EFSA expert panel states the simple dissimilar action “*occurs where the modes of action and possibly, but not necessarily, the nature and sites of toxic effects differ between the chemicals in a mixture, and one chemical does not influence the toxicity of another*” [123]. Based on the concept that toxic effects resulting from response addition would not be expected if no toxicity would occur from any of the single components of the mixture and given the low levels of pesticide residues in food, it was assumed that “… response-additive toxicity will rarely if ever occur from pesticide residues in food”. In contrast, when in a mixture, individual chemicals share the same mechanism of action, differing only in their potencies; we refer to this as dose addition, also known as simple similar action [113]. Finally, when one or more compounds interact in a mixture, we speak of interaction. The mechanistic basis of the interaction can be at the chemical, physico-chemical, or biological level. Thus, we can observe an interaction between two chemicals in a mixture or an interaction in either the toxicokinetic or toxicodynamic phase in a living organism. However, we need to distinguish between two types of interaction: synergistic (also referred as synergy, potentiation, or supra-additivity), when the combined effects of two or more interacting chemicals is either greater than that predicted based on dose addition or response addition; antagonistic (also called sub-additivity or inhibition), when the combined effects are lower than the individual chemical effect [113]. The basic assumption for the cumulative/combined risk assessment is dose addition, which considers compounds with similar mechanisms of action, or the same target organ [113,124,125]. Dose additivity has also been found for compounds with different mechanisms of action but displaying similar downstream in vivo effects, often indicated by “having effects on the same target organ” [103,113,126]. For this reason, the dose additivity assumption can be considered protective for human health assessments [113].

Many chemicals with anti-androgenic actions have been shown to act together in combination, producing effects at doses that individually are not associated with any observable responses [103,127,128,129,130,131,132]. Anti-androgens are compounds that can act on male sexual development but with different modes of action, such as the inhibition of androgen hormone biosynthesis or blocking of receptor-mediated signaling [112,133,134]. Chemicals with estrogenic action can also disturb the development of male reproductive organs [132,135,136,137,138,139,140], but little is known about the effects of mixtures of estrogenic and anti-androgenic chemicals [132].

Although great attention has been paid to EDC exposure, studies on human health are limited, especially those involving the general population. Different studies, focused on specific occupations related to pesticides belonging to EDCs, found an increased risk of prostate cancer in pesticide-related occupations such as pesticide applicators [115,141] and pesticide manufacturing workers [115,142]. Investigation of the role of pesticides in PC is complicated because of the need to obtain information on exposure to specific individual pesticides (in terms of type and time exposure), to track changes in pesticide use, and, because prostate cancer is so common later in life, to consider whether pesticides are associated with clinically significant or aggressive disease. In 2015, Lim et al. [115] conducted for the first time a meta-analysis of Persistent Organic Pollutants (POPs), such as polychlorinated biphenyls (PCBs) and organochlorine pesticides (OCPs), in terms of POP level and risk of prostate cancer in the general population [115]. POPs belong to EDCs, and they can be present in several foods [115]. In this study, Lim et al. examined the prostate cancer risk associated not only with each individual compound but also mixtures of them [115]. Due to the difficulty and high expense of human cohort studies, human epidemiological studies were conducted as a case-control study design, which is less costly and less time-consuming; however, with case-control studies, it is difficult to establish the timeline of exposure to disease outcomes [78]. Despite this, a case-control study showed an inverted U-shape association between oxychlordane, an organochlorine pesticide, and prostate cancer risk as well as linear positive associations between several PCBs and the risk of prostate cancer [78,143,144]. Another case-control study conducted in 2015 showed a positive association between plasma oxychlordane levels and metastatic prostate cancer risk in Norwegians [78,145]. Moreover, a positive association was found between high lindane exposure among pesticide applicators and a positive history of prostate cancer [146]. Lindane is an organochlorine insecticide that has a stable structure and is able to persist in the environment for a long time. Several years later, another case-cohort study using prospective cohort data was conducted to evaluate the association between serum concentrations of POPs and the risk of prostate cancer in the Korean population [78]. Lim et al. studied serum concentrations of 32 PCB congeners and 19 OCPs in 110 people diagnosed with prostate cancer [78]. This study showed that the serum levels of PCB156, PCB167, PCB153, and PCB180 were positively and significantly associated with the incidence risk of prostate cancer [78]. In particular, among PCBs, the strongest positive association with prostate cancer risk was shown only in the moderately chlorinated group, but an increased risk of prostate cancer incidence was also observed for highly chlorinated PCBs, biologically persistent PCBs such as CYP1A and CYP2B inducers, and the sum of non-dioxin-like PCBs [78]. The mechanism by which POPs can act on the prostate is unclear; hence, to understand how POPs can act, several experiments using cell lines, animals, and toxicological studies were performed, suggesting different mechanisms of action of various organochlorine pesticides (OCPs) on the development of PC [146,147,148]. An in vitro study performed on human metastatic prostate carcinoma (LNCaP) cells showed that dioxin-like coplanar PCBs interfere with androgenic pathways [78,149]. The reduced T-stimulated cell proliferation and inhibited prostate-specific antigen (PSA) secretion could be a function of the reduction of 5α-reductase activity and decreased DHT levels [115]. Another suggested hypothesis for carcinogenesis induced by POPs is the epigenetic mechanism; indeed, several studies have shown that global DNA hypomethylation is associated with exposure levels of POPs [78,150,151].

It is well known that a lag can occur between the time of EDC exposure and the manifestation of a disorder [1,152]. In experimental animals, it has been shown that exposure to mixtures of EDCs can induce adverse reproductive effects after birth, in puberty, and in young adulthood [121,127,132,152,153].

It has been demonstrated that developmental exposure to EDCs or elevated perinatal levels of estrogens can induce hyperplasia of the prostate epithelium in several animal studies [154,155]. Although most of the studies focused on chemicals with estrogenic modes of action, it has been shown that early anti-androgen exposure may increase the risk of pre-cancerous lesions in the prostate [152,154]. An epidemiological study showed associations between short anogenital distance and an increased prostate cancer risk [154,156], strengthening the hypothesis that prostate cancer risk may be increased by early anti-androgen substance exposure [154]. The influence of anti-androgenic chemicals on prostate development may differ from the influence of estrogenic chemicals, and it is important to compare the effects of anti-androgenic chemicals versus estrogenic chemicals on prostatic development to clarify the potential perinatal origins of prostate cancer [154]. Boberg and colleagues performed two different studies using a mixture of 13 anti-androgenic and estrogenic chemicals, with perinatal and pre-puberty exposure [152,154]. The mixture was based on human high-end exposure in order to reflect a realistic human intake and included eight predominantly anti-androgenic compounds, four estrogenic substances, and paracetamol, which has been shown to possess anti-androgenic effects in developing rats but has multiple additional actions, including prostaglandin synthesis inhibition [154]. The total mix of 13 anti-androgenic and estrogenic compounds induced histological changes at pup day (PD) 55, with an increased relative epithelial area and an increase in the presence of small acini, with columnar epithelium and papillary growth. At PD 300, further epithelial hypertrophy and a cribriform pattern were seen [154]. This effect was probably induced by a decrease of mRNA levels of Erβ, since its activation leads to antiproliferative effects in the prostate [154,157]. In rats, the ventral prostate normally atrophies with aging, probably with a decrease in T levels [152]. Isling et al. [152] showed that after exposure during gestation (from gestation day (GD) 7 to GD 21) to a mixture of anti-androgenic and estrogenic compounds, there was a hyperplasia of the ventral prostate at 10 months of age. This hyperplasia was probably due to a decrease of epithelial atrophy, high scores for atypical hyperplasia, and an increase in the appearance of cribriform patterns [152]. Since spontaneous tumors of the prostate are a rare finding in most rat strains, hyperplasia can be considered a marker of precancerous lesions [152]. Moreover, atypical hyperplasia in the rat prostate may also progress to adenoma and carcinoma [152]. In humans, high-grade prostatic intraepithelial neoplasia-lesions are considered a likely precursor of prostatic adenocarcinoma [152]. These lesions show cell proliferations of ducts and acini [152,158]. Thus, it is presumable that rat prostate hyperplasia, after mixture exposure during development, can suggest an increase of prostate cancer risk in humans [152]. Moreover, a recent study performed by Riad et al. [120] on male Wistar rats showed the combined effect of two EDCs: butylparaben (BP) and triclosan (TSC). BP is a preservative, and it is contained in many personal care and pharmaceutical products [120]. BP is considered an estrogenic agent since it enhances aromatase activity and increases the synthesis of estrogen hormones and the depletion of T levels [120,159]. TSC is a potent antimicrobial agent that is widely used as preservative in personal care products, plastics, and fabrics [120]. TSC has weak estrogenic activity and potentially induces vitellogenin expression in males and delays hatching in females [120,160]. The authors showed that TSC alone and in combination with BP (TSC+BP) was able to induce a decrease of relative weight of the ventral prostate gland. Moreover, the treatment induced hormonal disturbances such as a decrease in T, LH, and FSH levels and of the T/LH and T/E2 ratio, which in turn determined the prostate weight reduction [120]. Another study performed by Gan and colleagues [39] showed that a combination of Nonylphenol (NP) and BPA has toxic effects on the human prostate cell line (RWPE-1) in a dose-dependent manner, inducing a decrease in cell viability. BPA is an organic synthetic compound that is used primarily to produce polycarbonate plastics and epoxy resins [161]. Because of incomplete polymerization and degradation of the polymers, BPA has been found to easily leach in microgram amounts into food and water [39,162]. It is well known that BPA, at very low doses (1–10 nM), induces prostate cancer cell migration, modulating the ion channel protein expression [163]. Moreover, one study observed an association between high levels of urinary BPA and prostate cancer [164]. NP is a primary degradation product of nonylphenol ethoxylate (NPEO), a major group of multipurpose nonionic surface agents used as pesticide emulsifiers, defoaming agents, softening agents, and finishing agents in the textile industry [39]. Exposure to NP and BPA is ubiquitous, such that NP was detected in 51% of the urinary samples from 394 adults; BPA has been detected in >93% in the United States [39,100,165]. It has been shown that NP enhances the gene expression of key regulators of the cell cycle in PNT1A cells and promotes the translocation from cytoplasm to nucleus of ERα [32]. Moreover, the synergistic pro-estrogenic activity of NP and BPA has been demonstrated when they are co-incubated with prostate cancer cells [3,39]. Most likely, there is a competitive binding between the two chemicals. Both BPA and NP can inhibit aromatase activity and are agonists and antagonists of ERs and ARs, respectively [39]. Recently, we demonstrated that two alkylphenols, NP and Octylphenol (OP), both alone and in mixture, significantly inhibit steroidogenesis in the male lizard *Podarcis siculus* [111]. Intriguingly, showed that an NP + OP mixture has major effects on P450 aromatase, which converts androgens into estrogens [109,165]; this is quite interesting, as P450 aromatase represents a balance molecule between androgen and estrogens levels [111]. This data confirms that exposure to multiple endocrine-disrupting chemicals with similar or different modes of action leads to “cocktail” effects, and the combined exposure can lead to additive and synergistic effects [111,166,167,168,169]. Moreover, in a very preliminary study, we used a mixture of NP and E2 on two different human prostate cell lines: normal human prostate cells (PNT1A) and LNCaP [170]. DBP is short-chain phthalate prepared from butanol [34,171,172,173]. It is commonly used in paints, inks, adhesives, insecticides, solvents, cosmetics, perfumes, and medications [34,174,175,176,177]; thus, the human population is predominantly exposed to it [34,178,179]. Experimental studies on early gestation exposure to phthalates in rats have shown that they may display phthalate syndrome. These symptoms of this syndrome look like the effects of phthalate exposure in human males; it is characterized by the presence of seminiferous tubules with reduced diameter, hypospadias, cryptorchidism, reduced anogenital distance, and malformation of vas deferens, epididymis, seminal vesicles, and prostate glands [31,34,180,181]. The first data showed the preponderant effect of NP in all mixtures on PNT1A cells, whereas DBP overrides the NP effect on LNCaP cells. There was an increased cellular viability in LNCaP treated with NP and E2, indicating a possible synergistic effect between the compounds. On the contrary, DBP induced a decrease in cell viability. These effects were mediated by estrogen receptor pathways, mainly ERα [170].

## 2. Conclusions

Combined and cumulative studies on EDC mixtures are very difficult to perform, mostly due to the complexity in correctly interpolating the results, especially when combining two or more EDCs that have different modes of action. However, more and more studies show the role of different EDCs in the incidence of prostate diseases and in the increased risk of inducing prostate cancer in elderly men. Of note is the fact that exposure to EDCs, mostly with estrogenic action (but also elevated maternal E, pharmaceutical estrogens), during embryonic development, at the different windows of susceptibility, can induce permanent changes that determine the propensity to prostate pathologies later in the life. Through the collection of data presented in this review, we want to underline the urgency of increasing the research on EDC mixtures to better understand their role in and influence on prostate diseases.

## Abbreviations

ARs	Androgen Receptors
BOO	Bladder Outlet Obstruction
BP	ButylParaben
BPA	BisPhenol A
BPH	Benign Prostatic Hyperplasia
DBP	DiButylPhtalate
DHT	5α-DiHydroTestosterone
E	Estrogen
E2	17β-Estradiol
EDCs	Endocrine-Disrupting Chemicals
EFSA	European Food Safety Authority
ERs	Estrogen Receptors
GD	Gestation Day
LUTS	Lower Urinary Tract Symptoms
NOAELS	No Observed Adverse Effect Levels
NP	NonylPhenol
NPEO	NonylPhenol EthOxylate
OCPs	OrganoChlorine Pesticides
OP	OctylPhenol
PC	Prostate Cancer
PCBs	PolyChlorinated Biphenyls
PD	Pup Day
PND	Post Natal Day
POPs	Persistent Organic Pollutants
PRLR	Prolactin Receptor
PSA	Prostate-Specific Antigen
T	Testosterone
TSC	Triclosan
UGS	UroGenital Sinus
UNEP	UN Environment Programme
WHO	World Health Organization
